# Teprotumumab for treating active thyroid eye disease: A meta-analysis

**DOI:** 10.1097/MD.0000000000042966

**Published:** 2025-06-27

**Authors:** Xiangguo Cong, Leilei Pei, Honglei Hu

**Affiliations:** a Department of Endocrinology, The Affiliated Suzhou Hospital of Nanjing Medical University, Suzhou Municipal Hospital, Suzhou, China; b Department of Endocrinology, Sunshine Union Hospital, Weifang, China; c Department of Endocrinology, Shandong Zibo Central Hospital, Zibo, China.

**Keywords:** meta-analysis, teprotumumab, therapy, thyroid eye disease

## Abstract

**Background:**

Thyroid eye disease (TED) is a disabling, organ-specific autoimmune disease that is a global health concern. Recently, certain biological agents have demonstrated unique advantages for the treatment of TED. Teprotumumab is an emerging biological agent used for TED treatment. This study assessed whether teprotumumab can serve as an effective and safe treatment for active TED through a meta-analysis of the literature.

**Methods:**

We searched 4 databases (PubMed, The Cochrane Library, Web of Science, and Embase) for randomized controlled trials regarding the treatment of Graves’ ophthalmopathy by teprotumumab by March 31, 2024. We screened the literature library and extracted the data according to the inclusion and exclusion criteria.

**Results:**

Our study included 5 articles that involved 411 cases. Significant differences were reported in the change from baseline in proptosis (proptosis vs baseline), diplopia response at week 24, and clinical activity score of 0 or 1 at week 24 in the teprotumumab versus placebo group. The teprotumumab group reported no significant risk of adverse events or serious adverse events during the intervention.

**Conclusion:**

Teprotumumab significantly decreased proptosis and clinical activity score and improved diplopia response in patients with TED, with fewer adverse effects. Therefore, it is a promising biological agent. However, this conclusion should be further validated by high-quality, long-term randomized controlled trials with large sample sizes.

## 1. Introduction

Thyroid eye disease (TED), also called Graves’ ophthalmopathy (GO), or ophthalmopathy associated with thyroid,^[1]^ is mostly observed in Graves’ eye disease and accounts for approximately 90% of patients with TED. Patients with Hashimoto thyroiditis, hypothyroidism, and normal thyroid function also suffer from these diseases. TED is an autoimmune disease featuring infiltrative lesions in the posterior and periorbital tissues, and is the most prevalent orbital-related disease in adults.^[2]^ The primary manifestations include eyelid retraction, exophthalmos, diplopia, orbital pain, eyelid swelling, and lagophthalmos. Complications such as impaired vision, corneal ulceration, and dysthyroid optic neuropathy (DON) may be observed in severe cases and may seriously affect the quality of life of patients.^[3]^

Treatment for TED includes medication, orbital radiotherapy, and decompression surgery, among which medication mainly includes local anti-inflammatory agents, glucocorticoids, conventional immunosuppressive agents, and novel biological agents.^[4]^ Risk factors such as smoking and hypercholesterolemia should also be controlled. Surgery may be required for patients with severe conditions.^[5,6]^ However, TED is intractable and some drugs do not show satisfactory efficacy, resulting in an unfavorable prognosis that often requires multidisciplinary care. At present, the first-line treatment specific to moderate and severe TED is intravenous glucocorticoid pulse therapy; however, such therapy does not show satisfactory prognosis improvement and presents hormone-related side effects. The second-line treatment is glucocorticoid pulse therapy or a combination of orbital radiotherapy and other immunomodulatory agents. Teprotumumab is a monoclonal antibody that blocks the immune response to TED by specifically binding to the extracellular region of IGF receptor type 1 (IGF-1R), which may restrict the binding of IGF-I and IGF-II to their receptors and inhibit the signaling pathways of the receptor complexes of IGF-IR and thyroid-stimulating hormone receptor.^[7,8]^ Teprotumumab blocks the activity of the IGF-1R, which has a role in several physiological processes, including growth, metabolism, and inflammation.^[9]^ Teprotumumab is recommended as the drug of choice for the treatment of patients with active moderate-to-severe TED with proptosis and/or diplopia in a position statement developed by experts from the American Thyroid Association and the European Thyroid Association.^[10]^ Therefore, this meta-analysis aimed to conduct a systematic assessment of the effectiveness and safety of teprotumumab for TED treatment of TED.

## 2. Materials and methods

### 2.1. Search strategy

We searched 4 databases (PubMed, The Cochrane Library, Web of Science, and Embase) by March 31, 2024, with the search items of teprotumumab and GO.

### 2.2. Inclusion and exclusion criteria

#### 2.2.1. Inclusion criteria

(1) Participants

Randomized controlled trials (RCTs) of patients with active GO (clinical activity score [CAS] ≥ 4).

(2) Intervention

This meta-analysis integrated articles on teprotumumab administered for at least 24 weeks in patients with GO.

(3) Outcome measures

Proptosis versus baseline, diplopia response at week 24, CAS of 0 or 1 at week 24, and adverse effects (i.e., adverse event [AE] and serious adverse event [SAE] during intervention). AEs during the intervention phase were those occurring in more than 5% of patients in the teprotumumab group and more frequently in the teprotumumab group than in the placebo group. Serious adverse events included any event requiring hospitalization.

#### 2.2.2. Exclusion criteria

(1) Incomplete literature or those with duplicate publications; (2) meta-analyses, letters, case reports, meeting abstracts, etc; (3) articles incapable of obtaining the full text and the needed outcomes; (4) nonhuman clinical trials and non-RCTs regarding teprotumumab therapy; (5) articles of GO that are unclearly diagnosed or combined with other diseases.

### 2.3. Study selection and data extraction

Two authors screened the respective literature titles and abstracts according to the inclusion and exclusion criteria to obtain the full texts of those meeting the criteria. They independently extracted useful data. A third investigator was required to deal with disagreements between them.

### 2.4. Risk of bias evaluation

Two authors evaluated the risk of bias using the Cochrane Collaboration’s risk-of-bias evaluation kit, which mainly covers 6 items: (1) selective report (report bias); (2) incomplete outcome information (attrition bias); (3) outcome evaluation blinding (detection bias); (4) participant and personnel blinding (performance bias); (5) allocation concealment (selection bias); (6) random sequence generation (selection bias); and (7) other biases.

### 2.5. Statistical analysis

Revman 5.3 (The Cochrane Collaboration) was used for the meta-analysis. Analysis was conducted using 95% confidence intervals (CI) and mean differences (MD). The *I*^2^ statistic was used to evaluate statistical heterogeneity, and *I*^2^ < 50% and *P* > .1 reported no statistical significance. Data pooling received a fixed-effect model analysis. *I*^2^ > 50% and *P* < .1 reported substantial heterogeneity, and in this case, the random-effect model was adopted. A sensitivity survey helped evaluate the influence of individual trials on the pooled effect by sequentially omitting the respective trials. Forest plots represent the graphic data. Funnel plots were used to test the publication biases (PB).

## 3. Results

### 3.1. Search results

**Figure 1.**
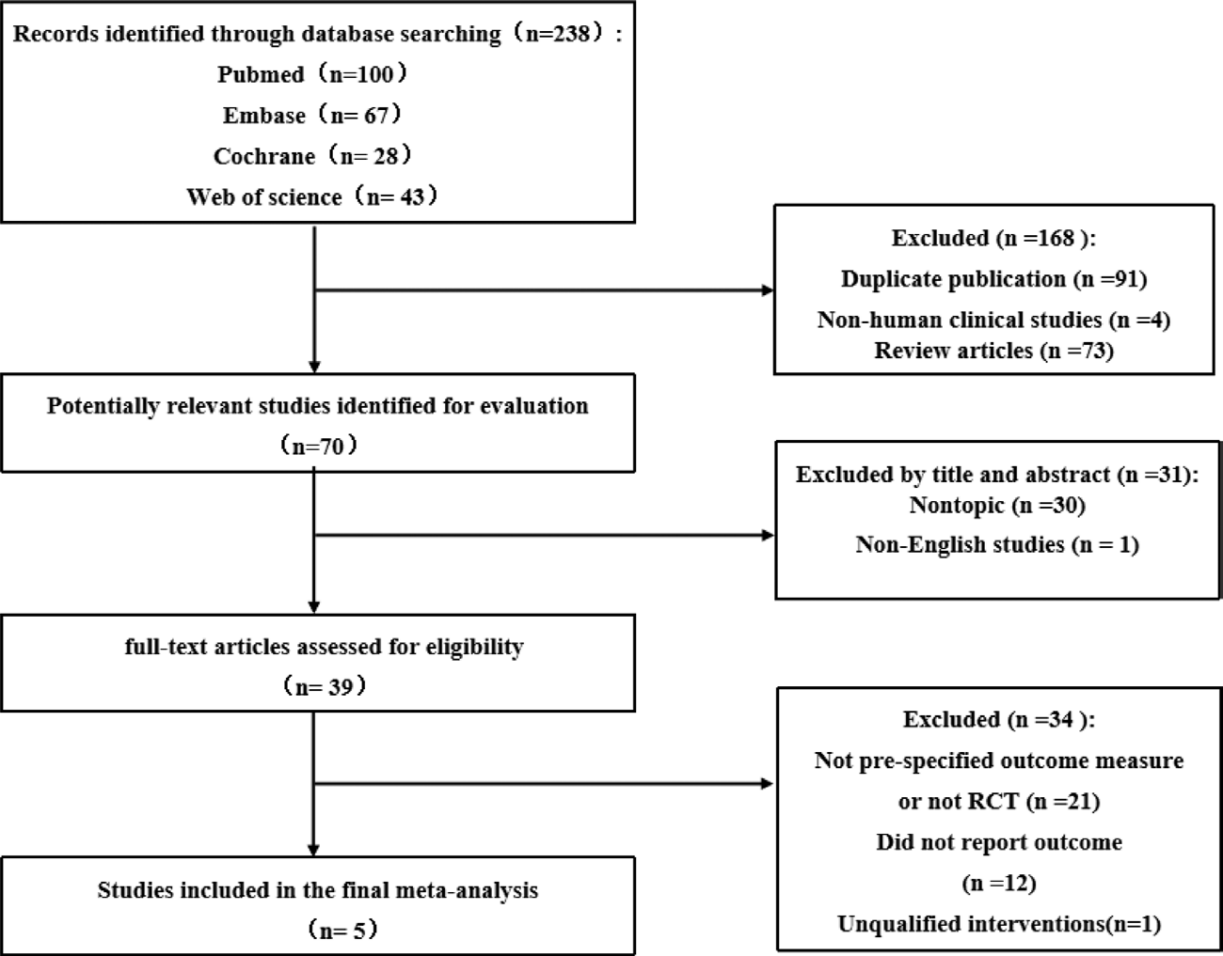
Flow chart of the literature search process.

We retrieved the existing literature to obtain 238 eligible articles and finally identified 5 articles here.^[11–15]^ Figure 1 shows a selection flow diagram.

### 3.2. Article characteristics and quality evaluation

**Figure 2.**
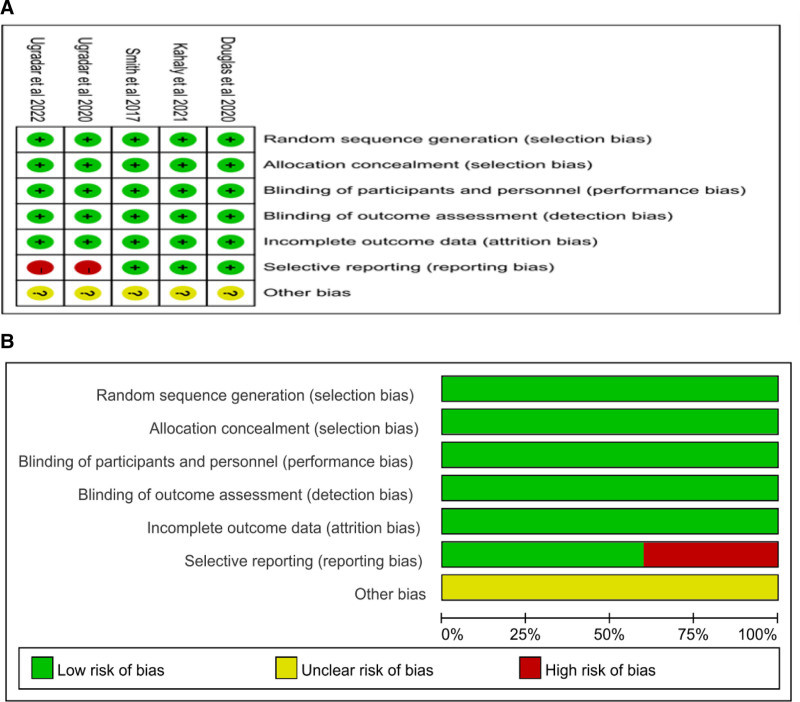
(A) Risk of bias summary: review the author’s judgements about the respective risk of bias item for each involved study. (B) Risk of bias graph: review researchers’ judgements about the respective risk of bias items presented as percentages across all involved articles.

Five studies published between 2017 and 2022 were selected, with a total of 411 subjects. The treatment and control groups included 201 and 210 patients, respectively. Of the 5 articles, one with the least number of study subjects involved 10 cases and one with the largest number of study subjects involved 87 cases. Table 1 presents the basic characteristics of the study. The Cochrane risk bias evaluation tool was used to evaluate the quality of the literature (Fig. 2).

**Table 1:** The characteristics of the involved observational.

No	Study	Intervention	Age (years)	Female (%)	Duration of TED (months)	Number of smoker (n)	CAS	Baseline proptosis (mm)	Diplopia at baseline (%)	Follow-up duration (weeks)
1	Smith et al^[11]^	T: Teprotumumab (n = 42) C: placebo (n = 45)	51.6/54.2	65/82	4.7/5.2	11/18	5.1/5.2	23.40/23.10	90/69	24
2	Douglas et al^[12]^	T: Teprotumumab(n = 41) C: placebo (n = 42)	51.6/48.9	71/74	6.2/6.4	9/8	5.1/5.3	22.62/23.20	68.3/66.7	24
3	Ugradar et al^[13]^	T: Teprotumumab(n = 10) C: placebo (n = 12)	50/59	60/83	5.8/6.0	2/4	5.0/5.4	24.20/22.80	100/66.7	24
4	Kahaly et al^[14]^	T: Teprotumumab(n = 84) C: placebo (n = 87)	51.5/51.4	69/77	5.7/6.8	20/26	5.1/5.3	23.02/23.15	79/68	24
5	Ugradar et al^[15]^	T: Teprotumumab(n = 24) C: placebo (n = 24)	48.5/50.29	58/58	6.1/6.4	6/4	5.1/5.5	22.20/22.70	91.7/62.5	24

CAS = clinical activity score, TED = thyroid eye disease.

In 5 articles, random sequence generation was clear (100%), and all articles (100%) were allowed to be adequately hidden. In addition, double-blinding was suggested for all articles (100%). Among the 5 articles, all articles (100%) had clear results, but except for 2 articles that had to report biases as impacted by the post hoc analysis, all articles had no idea about other biases as impacted by the small sample size. In addition, according to the analysis, the articles involved did not present significant PB.

### 3.3. Efficacy outcomes

#### 3.3.1. Effect of the proptosis versus baseline

**Figure 3.**
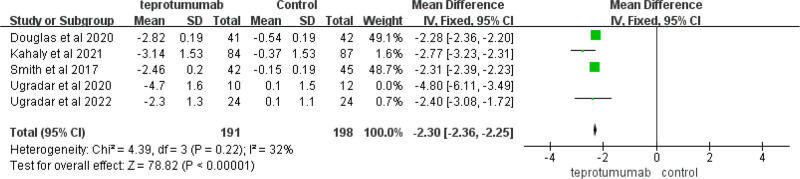
Effect on the change from baseline in proptosis in articles comparing teprotumumab to placebo control.

The study focused on the evaluation of the effect of teprotumumab on proptosis versus baseline in 5 articles with 411 participants. Based on the heterogeneity analysis, we excluded one paper that was highly heterogeneous when performing reanalysis. The remaining 4 studies^[11,12,14,15]^ showed little heterogeneity after consolidation (*Chi* ^2^ = 4.39, *P* = .22, *I*^2^ = 32%). Finally, we adopted a fixed-effects model. Proptosis changed significantly from baseline in the studies of teprotumumab versus placebo (*MD*, −2.30 [95% *CIs*, −2.36 to −2.25], *P* < .00001). In addition, sensitivity analysis was conducted on the included literature, and no single study greatly interfered with the meta-analysis results, indicating that the included studies were stable (Fig. 3).

#### 3.3.2. Effect of the diplopia response at week 24

**Figure 4.**
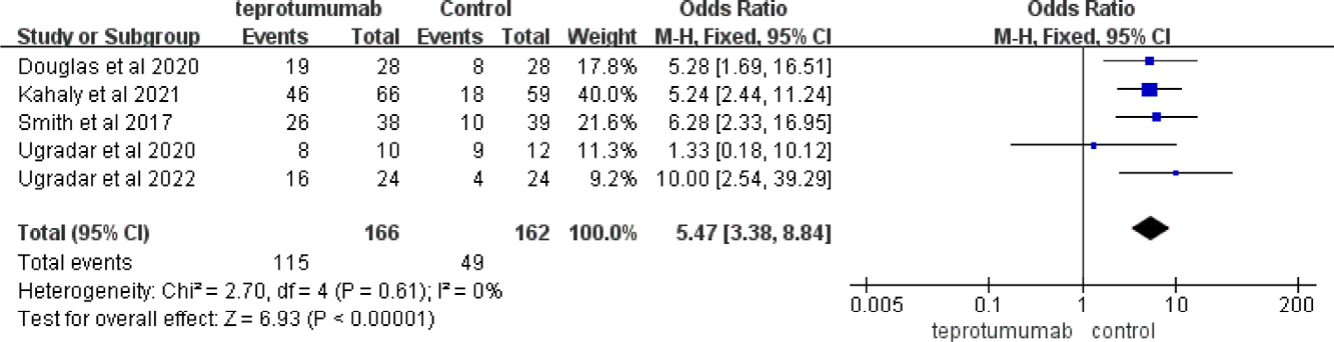
Effect on the diplopia response at week 24 in articles comparing teprotumumab to placebo control.

Five articles (n = 411) showed the effect of diplopia response at week 24 between the teprotumumab treatment groups (teprotumumab) and the control (control). In the analysis of the 5 studies, there was no heterogeneity within the groups (Chi^2^ = 2.70, *P* = .61, *I*^2^ = 0%), and the effect on the diplopia response at week 24 was significant compared with teprotumumab in the control (*MD* = 5.47, 95% *CI* [3.38,8.84], *P* < .00001) (Fig. 4).

#### 3.3.3. Effect of the CAS of 0 or 1 at week 24

**Figure 5.**
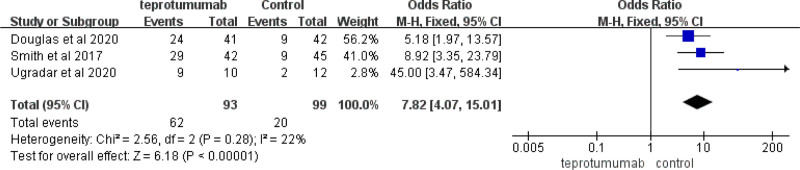
Effect on the CAS of 0 or 1 at week 24 in articles comparing teprotumumab to placebo control. CAS = clinical activity score.

Three articles^[11–13]^ explained how teprotumumab treatment affected the effect of CAS of 0 or 1 at week 24 (n = 192). These articles did not show obvious heterogeneity (*Chi* ^2^ = 2.56, *P* = .28, *I*^2^ = 22%). There were obvious changes in the treatment with teprotumumab versus placebo (*MD*, 7.82 [95% *CIs*, 4.07 to 15.01], *P* < .00001) (Fig. 5).

### 3.4. Adverse events

#### 3.4.1. AEs during the intervention

**Figure 6.**
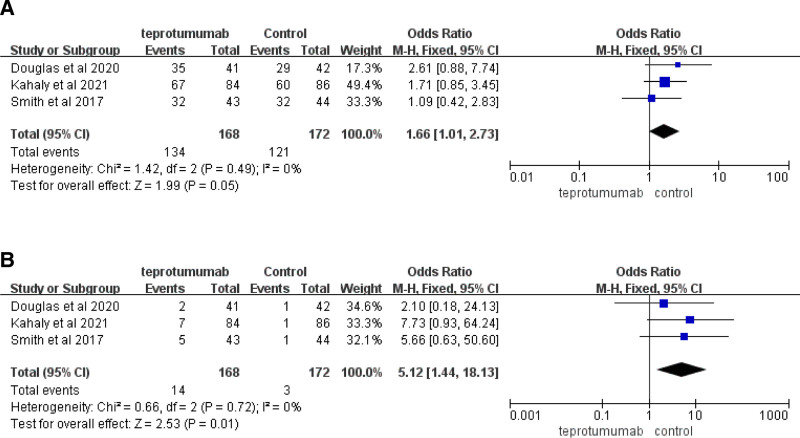
(A) Incidence of adverse events during the intervention in articles comparing teprotumumab to placebo control. (B) Incidence of serious adverse events during the intervention in articles comparing teprotumumab to placebo control.

AE include muscle spasms, alopecia, nausea, fatigue, diarrhea, headache, hearing impairment, dysgeusia, stomatitis, amenorrhea, dizziness, cough, and upper abdominal pain. Three articles (n = 340) revealed a difference between the teprotumumab and control groups regarding AEs during the intervention (*MD*, 1.66 [95% *CIs*, 1.01–2.73], *P* = .05) but did not confirm any obvious heterogeneity (*Chi*^2^ = 1.42, *P* = .49, *I*^2^ = 0%) (Fig. 6A).

#### 3.4.2. Serious AE during the intervention

SAEs include optic neuropathy, diarrhea, inflammatory bowel disease, escherichia sepsis, Hashimoto encephalopathy, and urinary retention. Three articles (n = 340) indicated SAEs during the intervention between Teprotumumab and the Control (*MD*, 5.12 [95% *CIs*, 1.44–18.13], *P* = .01). No obvious heterogeneity was identified between the teprotumumab and placebo (*Chi*^2^ = 0.66, *P* = .72, *I*^2^ = 0%) (Fig. 6B).

There was one case of SAEs, including infusion reaction, pneumothorax, visual-field defect, Hashimoto encephalopathy, and inflammatory bowel disease.

### 3.5. Sensitivity analysis evaluation and publication bias

**Figure 7.**
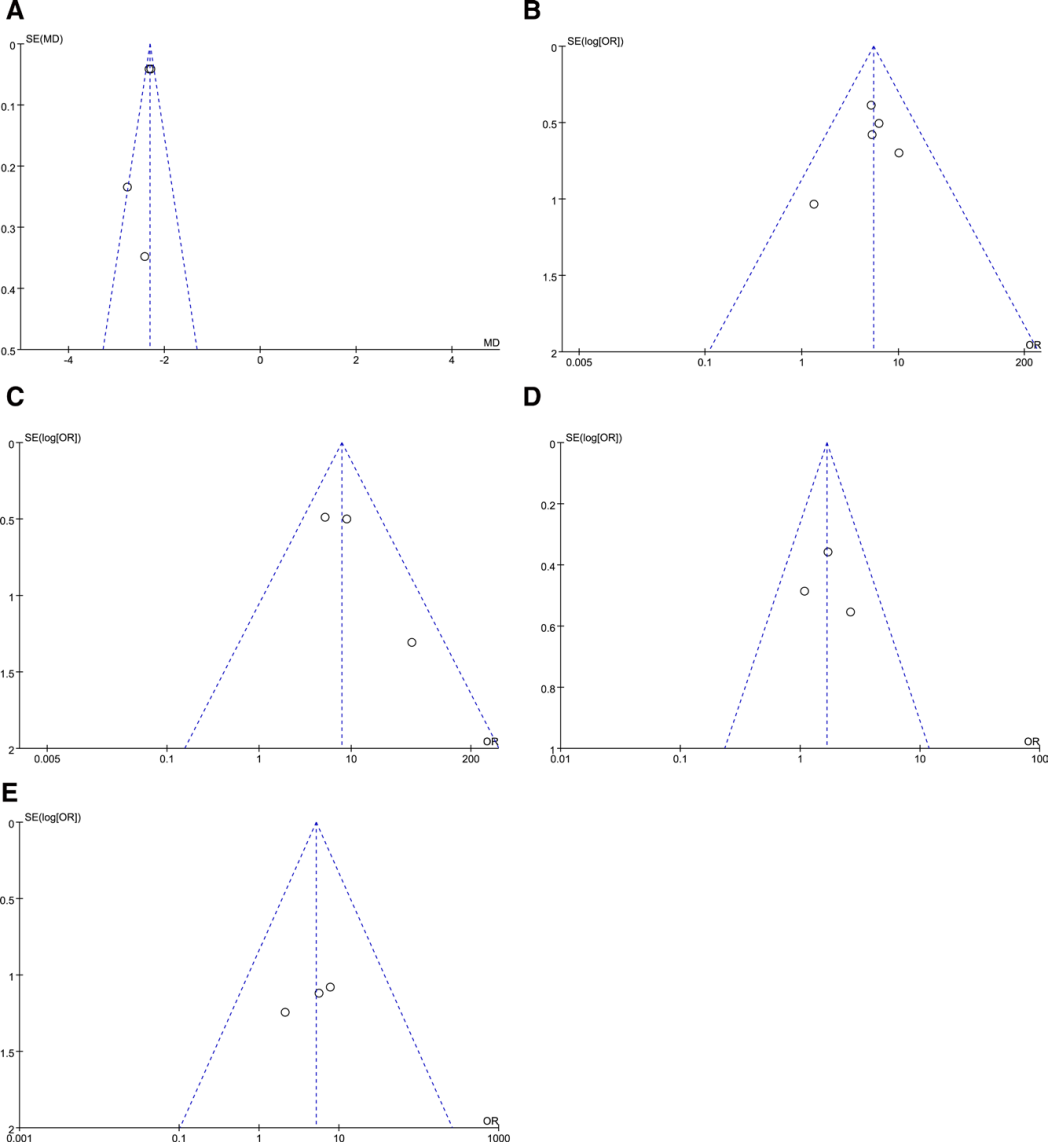
Funnel plots of the effects of teprotumumab treatment on (A) the change from baseline in proptosis, (B) the diplopia response at week 24, (C) the clinical activity score (CAS) of 0 or 1 at week 24, (D) adverse event during the intervention, and (E) serious adverse event during the intervention.

According to the sensitivity analysis, no articles exerted an obvious interfering influence on the meta-analysis results; that is, these articles were strongly stable. Funnel plots systematically explained the effectiveness and AEs indicators, including proptosis versus baseline, diplopia response at week 24, CAS of 0 or 1 at week 24, and adverse effects (AE and SAE during intervention) (Fig. 7A–E). Funnel plots (A) to (E) showed a symmetrical distribution, involving most of the tested scatter points, thereby revealing the insignificant possibility of PB.

## 4. Discussion

TED is a progressive autoimmune disease, and only a few epidemiological studies have been conducted. The annual incidence of TED in the general population is approximately 3.3/100,000 (women) or 0.9/100,000 (men).^[16]^ TED may be classified according to disease severity as mild, moderate-to-severe, or sight-threatening (very severe). Meanwhile, CAS servers for staging disease activity. The clinical manifestations of TED vary with age, ethnicity, and sex. Women tend to have an earlier age of onset, but their disease severity is generally lower than that in men.^[17]^ Patients aged > 60 years tend to develop more severe TED, which often manifests as extraocular muscle enlargement.

We are now moving away from Rundle curve. New data suggests TED is a chronic condition and never becomes inactive. There is a study by Ugradar et al^[18]^ that shows IGF-1R overexpression in TED persists into the chronic phase. Further, a study by Cockerham et al,^[19]^ discusses the persistence of TED-related symptoms well into the chronic phase. Only 3% to 5% of patients with TED can develop very severe disease, manifested as vision-threatening corneal ulcers or DON.^[20]^ In addition to its possible impact on appearance and vision, TED may seriously affect the quality of life of patients.^[21]^ Currently, great progress has been made in the exploration of TED pathogenesis. Nevertheless, it remains poorly understood and may be associated with immunity, genetics, environment, and other factors. The pathogenic targets of TED include the thyrotropin receptor (thyroid-stimulating hormone receptor), insulin-like growth factor 1 receptor (IGF-1R), interleukin-6 and interleukin-17A receptors, and neonatal Fc receptor, among others.^[22,23]^ Clinical TED treatment is poorly developed and places a great burden on both patients and society. For the treatment of active TED, glucocorticoid therapy or orbital radiotherapy regimens that are currently recommended may improve inflammation to some extent but are ineffective for proptosis and diplopia and may result in certain side effects.^[24,25]^ To date, no treatment has curbed the natural course of TED.

IGR-1R has received extensive attention with investigations into TED pathogenesis and is a popular target for anticancer drugs. It is expressed on the surface of a variety of cell membranes, which activates a series of downstream signaling pathways upon binding to ligands, which may in turn affect cell proliferation, differentiation, and apoptosis.^[26]^ TED orbital tissue of patients with TED has much higher IGF-1R levels than that of the normal population.^[27–29]^ Upon activation, IGF-1R can initiate autoimmune responses in the body, resulting in the generation of many extracellular matrix and inflammatory factors and swelling of the eye muscles and adipose tissue, causing eyelid retraction and protrusion of the eyeball. Therefore, blocking IGF-1-to-IGF-1R binding may inhibit the activation of IGF-1R-mediated signaling pathways, which would reduce the expression of downstream inflammatory factors, thus preventing the secretion of hyaluronic acid and inflammatory factors by orbital fibroblasts.^[30,31]^ The use of biological agents against pathogenic targets of TED has become a promising approach for treating TED. As a fully human monoclonal antibody targeting IGF-1R that inhibits IGF-1R-mediated signaling pathways by binding to the IGF-1R complex, teprotumumab alleviates inflammation and improves symptoms, such as proptosis and diplopia.

The CAS was introduced by Mourits in 1989 and includes the assessment of 7 factors: chemosis, eyelid swelling and erythema, conjunctival erythema, caruncular swelling, pain in primary gaze, and pain with ocular movement. The following items were added to the 7-item scale to produce a 10-item CAS: ≥2 mm increase in proptosis, decrease in visual acuity, and ≥8-degree decrease in eye movements (all 3 items refer to the last 1–3 months).^[32]^ A score of ≥3 on a 7-point scale indicates active TED when anti-inflammatory therapy is effective in suppressing the inflammatory response, whereas treatment becomes significantly less effective once fibrosis develops. The CAS of the cases included in this meta-analysis was above 4/7 (7-point scale). The teprotumumab-treated group had a higher proportion of CAS (0 or 1) than did the placebo group.

Proptosis is the main clinical manifestation in patients with TED due to orbital fat inflammatory edema and extraocular muscle hypertrophy,^[33]^ and approximately 60% of TED patients have proptosis. These patients were also more likely to develop lagophthalmos.^[34]^ According to a meta-analysis, compared to the control, teprotumumab significantly reduced proptosis at 24 weeks. In a study by Ugradar et al^[15]^ in 2022, 63% of the patients whose proptosis did not improve (≥2 mm from baseline) after 12 weeks of teprotumumab administration showed significant clinical improvement at 24 weeks after continued treatment, suggesting that there was variability in the time to efficacy of teprotumumab and that some patients may require longer treatment periods.

The main cause of diplopia in patients with TED is restrictive strabismus caused by extraocular muscle degeneration, particularly in the inferior and medial rectus muscles, which can be unilateral or bilateral, iplopia. As the most intractable clinical manifestation of TED, remarkably affects the quality of life of patients. This meta-analysis suggested that diplopia was significantly alleviated following teprotumumab treatment compared to placebo, while available evidence suggests that biological agents such as rituximab and tocilizumab were less effective against diplopia,^[35,36]^ making this effect a likely unique advantage of teprotumumab.

Recent studies have also suggested that teprotumumab may be used in the treatment of stable chronic TED, with previous experience suggesting that immunotherapy is less effective after fibrosis. A retrospective study of 31 TED patients with a disease duration of >2 years and a CAS of ≥3 by Ugradar^[37]^ showed improvement in proptosis, orbital soft tissue volume, diplopia, inflammation, and strabismus in TED patients after teprotumumab treatment. DON, the most serious complication of TED, has been reported to have been successfully treated with teprotumumab in patients with DON who have undergone unsuccessful intravenous glucocorticoid and radiation therapies.^[38–40]^

During clinical treatment, potential adverse effects must be considered when administering all drugs. In the analyzed studies, representative AEs following the application of teprotumumab were hyperglycemia, muscle spasm, alopecia, nausea, fatigue, headache, dry skin, hearing impairment, and cough. Most of the symptoms were mild, did not affect treatment, and did not require special attention. Hyperglycemia was the only drug-related AE, but the blood glucose level was controlled with appropriate treatment. Hearing impairment occurs in about 7% to 10% of cases and is mostly remissionable, although hearing impairment is a worrisome AE, and its mechanism and reversibility need to be further investigated.^[41]^ In a study by Smith et al,^[11]^ 5 of 43 patients treated with teprotumumab experienced SAEs. The investigators classified diarrhea and mental confusion as “possibly drug-related” and other SAEs as “unrelated.” The 2 SAEs reported by Douglas^[12]^ were pneumothorax and an infusion reaction leading to drug discontinuation. The investigators judged pneumothorax to be unrelated to the trial drug. No fatalities were reported in any of the studies.

This study had some limitations. First, despite the high quality of the studies included in this meta-analysis, only 5 studies were included, and some of them had small sample sizes. Second, the drugs in the control groups of the included studies were limited to placebo, and no comparison was made with first-line drugs for the treatment of TED, such as glucocorticoids. Finally, several of the included studies were from similar research teams, and there may have been some consistency in study design, methodology, or data processing, with potential implications that may exist, and more high-quality, different-team studies will be included in future studies to further enhance the generalizability and stability of the findings. Given the relatively high price of teprotumumab, the cost-effectiveness of the treatment should be considered during trial design.

## 5. Conclusion

This meta-analysis showed that teprotumumab could safely and effectively treat patients with active TED. However, the high price of teprotumumab and its availability only in the United States also limit its clinical application. Few clinical studies have been conducted to date, and the sample sizes have generally been too small. High-quality RCTs with large sample sizes comparing teprotumumab with drugs such as glucocorticoids are required to more accurately elucidate the therapeutic effect and safety of teprotumumab for TED.

In conclusion, teprotumumab is an emerging biological agent with significant effects on TED treatment. Attention should also be paid to the adverse effects of teprotumumab, which should be closely monitored.

## Acknowledgments

We thank the authors of this article for their contributions to this study. Language editing was performed by Editage (https://www.editage.com).

## Author contributions

**Conceptualization:** Xiangguo Cong, Leilei Pei, Honglei Hu.

**Data curation:** Xiangguo Cong, Leilei Pei, Honglei Hu.

**Formal analysis:** Xiangguo Cong, Leilei Pei.

**Funding acquisition:** Xiangguo Cong.

**Investigation:** Xiangguo Cong, Honglei Hu.

**Methodology:** Xiangguo Cong.

**Project administration:** Xiangguo Cong, Honglei Hu.

**Resources:** Xiangguo Cong.

**Software:** Xiangguo Cong, Leilei Pei, Honglei Hu.

**Supervision:** Xiangguo Cong.

**Validation:** Xiangguo Cong.

**Visualization:** Xiangguo Cong.

**Writing – original draft:** Xiangguo Cong, Leilei Pei.

**Writing – review & editing:** Xiangguo Cong, Leilei Pei, Honglei Hu.
